# Recent advances in taste transduction and signaling

**DOI:** 10.12688/f1000research.21099.1

**Published:** 2019-12-17

**Authors:** Sue C. Kinnamon, Thomas E. Finger

**Affiliations:** 1 Rocky Mountain Taste & Smell Center, Department of Otolaryngology and Department of Cell & Developmental Biology, University of Colorado School of Medicine, Aurora, CO, 80045, USA

**Keywords:** taste bud, transduction, geniculate ganglion, purinergic transmission, serotonin, taste, signalling, nervous system

## Abstract

In the last few years, single-cell profiling of taste cells and ganglion cells has advanced our understanding of transduction, encoding, and transmission of information from taste buds as relayed to the central nervous system. This review focuses on new knowledge from these molecular approaches and attempts to place this in the context of previous questions and findings in the field. The individual taste cells within a taste bud are molecularly specialized for detection of one of the primary taste qualities: salt, sour, sweet, umami, and bitter. Transduction and transmitter release mechanisms differ substantially for taste cells transducing sour (Type  III cells) compared with those transducing the qualities of sweet, umami, or bitter (Type II cells), although ultimately all transmission of taste relies on activation of purinergic P2X receptors on the afferent nerves. The ganglion cells providing innervation to the taste buds also appear divisible into functional and molecular subtypes, and each ganglion cell is primarily but not exclusively responsive to one taste quality.

This review will focus on progress in the last few years in understanding transduction, coding, and specificity of the peripheral gustatory system starting with the cellular components of taste buds. Taste buds comprise 50 to 100 taste cells, which are specialized epithelial cells including signal transducing cells and glial-like supporting cells. The taste cells respond to taste substances in the saliva to generate a biological signal that then must be transmitted to the taste sensory nerves conveying the message to the nucleus of the solitary tract in the brain stem. This review will focus on recent discoveries in the processes of taste transduction and transmission, which significantly impact our understanding of this system.

In mammals, the sense of taste detects a wide variety of compounds which canonically fall into only five main sensory qualities (or modalities): sweet, umami (savory), bitter, sour, and salty. Each taste cell is most responsive to a single taste quality ^1^, and each taste bud contains one or more cells capable of responding to each of the taste qualities; that is, taste cells are “chemically tuned” whereas taste buds are not.

## Methodological advances

Two relatively new methodologies have been put to good effect in analyzing taste buds and their associated ganglia. Functional imaging of ganglion cells expressing genetically encoded activity sensors has allowed direct analysis of the breadth of tuning of inputs to the central nervous system (CNS) ^2, 3^, whereas single-cell transcriptomics has permitted molecular and functional classification of both taste cells and ganglion cells ^4– 8^. The implications of these and other recent studies are described below and summarized in Table 1.

**Table 1.** Reported molecular characteristics of taste cells and ganglion cells.

Taste quality	Sweet	Umami	Bitter	Sour	Salty
Ganglion cells	*Spon1*	*Cdh4?* ^a^	*Cdh13 ?* ^a^	*Penk*, *Htr3a*	*Egr2?* ^a^
Taste cells	*Sema7A*		*Sema3A*	*Pkd2L1*, *OTOP1*	

Data are from 6, 8– 11. ^a^The meta-analysis by Anderson and Larson ^11^ does not support Cdh4, Cdh13, and Egr2 as marking unique clusters. *Cdh13* marked two clusters that also express *Olfm3*. *Olfm3* is associated with several ganglion cell clusters but is never associated with *Penk* and so may mark cells innervating Type II cells ^4^ but not a particular subset of Type II cells. Factors in question are indicated by “?”.

## Transduction

**Figure 1.**
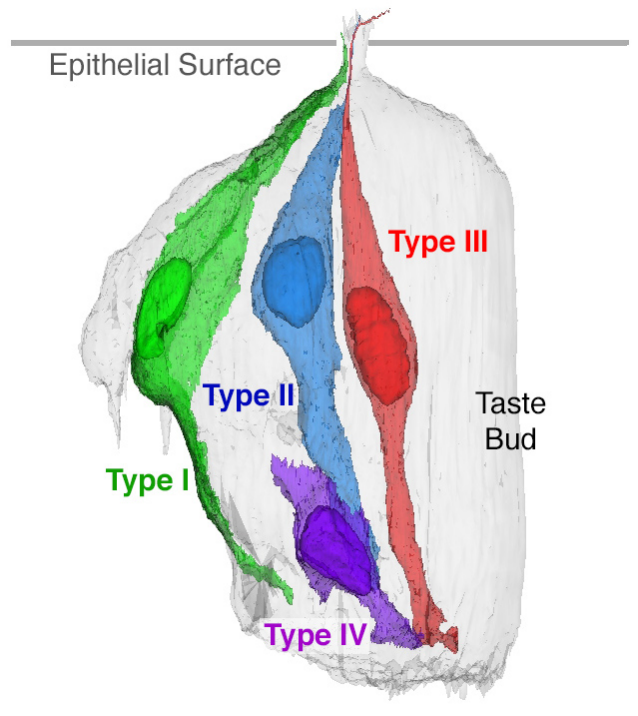
Cell types in taste buds. Four different morphological and molecularly distinct types of cells populate taste buds. Types II and III transduce different classes of tastes, whereas Type I cells are more glial-like. Type IV cells are the immature population, which develop into the other cell types over the span of a few days. Figure generated from data in 12.

The taste cells are divisible into four types characterized by both morphological and molecular features and given the names Type I, Type II, Type III and Type IV ( Figure 1). Type I cells are similar in many ways to astrocytes and Type IV cells are immature cells, whereas Type II and Type III cells serve as the transducing elements for different taste qualities.

Type II cells use G protein–coupled receptors for sweet (T1R2 + T1R3), umami (T1R1 + T1R3), or bitter (T2Rs) to initiate a transduction cascade, whereas Type III cells rely on ion channels for transduction of the ionic tastes of salty and sour. The receptors and downstream signaling cascade for the Type II cells (sweet, umami, or bitter) have been well described since the early part of this century ^13^ and involve a phospholipase C (PLC)-mediated cascade culminating in the activation of the Ca ^++^-responsive channels TRPM5 and TRPM4 ^14^ to depolarize the cell sufficiently to generate an action potential via voltage-gated Na ^+ ^channels (SCN2A, SCN3A, and SCN9A ^15^). Why axonless receptor cells should generate action potentials is of interest and is likely related to the release mechanism for neurotransmitter from Type II taste cells as described below.

Whereas early studies suggested that a single sweet taste receptor (T1R2 + T2R3) mediates all responses to sugars and sweeteners ^16^, recent studies suggest that other mechanisms also play a role for glucose-containing sugars but not for artificial sweeteners. Glucose transporters and the K _ATP_ channel, which are expressed in sweet-responsive (T1R3-expressing) taste cells ^17^, are involved in cephalic phase insulin release independent of the neural signal for sweet transmitted to the nervous system ^18^. The exact mechanism by which activation of the taste cells evokes insulin release is unclear but may involve humoral rather than neural signals.

### Sour

In 2006, Huang *et al*. ^19^ showed that sour detection depended on cells expressing PKD2L1—cells subsequently identified as a subset of Type III cells ^20^. In 2011, Horio *et al*. showed that PKD2L1 itself was not necessary for transduction of protons ^21^. Rather, transduction of sour involves permeation of H ^+^ through an apical ion channel ^22^ subsequently identified as OTOP1 ^6^. Using PKD2L1 as a molecular identifier for sour-responsive taste cells, Liman *and co-workers* ^7^ and Zhang *et al*. ^8^ went on to confirm OTOP1 as the necessary transduction channel underlying sour taste. The entering H ^+^ ions not only directly depolarize the taste cells but also block Kir2.1 K ^+^ channels ^23^, thereby amplifying the depolarization of the entering H ^+^ ions. The resulting depolarization triggers voltage-gated Na ^+^ channels (SCN2A ^15^) to generate action potentials ** that activate voltage-gated Ca ^++^ channels triggering the release of synaptic vesicles ^24^.

In keeping with the PKD2L1 cells being the sour-transducing cells, optogenetic driving of these cells evokes an aversive response ^25^. Curiously, another study ^26^ reported that optogenetic driving of the PKD2L1 population drives drinking behavior in thirsty mice. Why the mice should respond with drinking to a sensation of sour is still unresolved, although Zocchi *et al*. ^26^ suggest that a subset of PKD2L1-expressing cells may convey a specific “water taste” as is known for insects ^27– 29^.

### Salt

Historically, responsiveness to salt has been separated into amiloride-sensitive (AS) and amiloride-insensitive (AI) modalities ^30^. Confounding our understanding of salt taste is that low concentrations of salt are appetitive whereas high concentrations are aversive. Furthermore, while Na ^+^ is important perceptually for salt, other substances, not containing Na ^+^, also are salty-tasting. The multiple perceptual and chemical properties suggest that more than one transduction mechanism may be involved. Supporting this concept are the results from ^31, 32^. These studies argue that the molecular correlate of AS salt, the epithelial sodium channel (ENaC), underlies the appetitive qualities of Na ^+^ but that AI salt detection of high concentrations of Na ^+^ relies on a subpopulation of the bitter-responsive Type II cells and a subset of the sour-responsive Type III cells ^5, 32, 33^. Furthermore, a recent study ^34^ suggests that the AI salt transduction mechanism may directly involve Cl ^−^, but the actual mechanism remains elusive since known Cl ^−^ channel and transporter blockers have no effect on salt taste. Two recent works confirm a previous study ^35^ suggesting that AS-responsive taste cells fall into a unique class of taste cells not identified by the canonical reporters (TRPM5 for Type II and CAR4 for Type III ^32^ or PIRT for Types II and III ^34^). Further confounding the interpretation of these studies on Na ^+^ transduction is the finding that, compared with rodents, humans—who enjoy low salt and avoid high salt—do not have a large AS component of salt taste ^36^, although chimpanzees do have a minor AS component ^37^.

## Peripheral neurotransmission

Whatever means are used for transduction, the Type II and Type III cells ultimately must release one or more neurotransmitters to activate the afferent nerves. Activity-dependent release of ATP from Type II cells ^38, 39^ and 5-HT from Type III cells ^40^ was clearly demonstrated by several investigators, but whether other transmitters (for example, glutamate or acetylcholine) also may be involved remains unclear. Although ATP acting on neural P2X receptors was identified as a crucial transmitter for all tastes over a decade ago ^41^, only recently has the contribution of 5-HT acting on neural 5-HT _3_ receptors been elucidated in terms of transmission of sour taste ^42^. In addition, taste cells may directly release peptides such as glucagon-like peptide 1 (GLP-1) ^43, 44^. These additional transmitters may act to modulate adjacent taste cells ^45^ and activate afferent nerve fibers.

**Figure 2.**
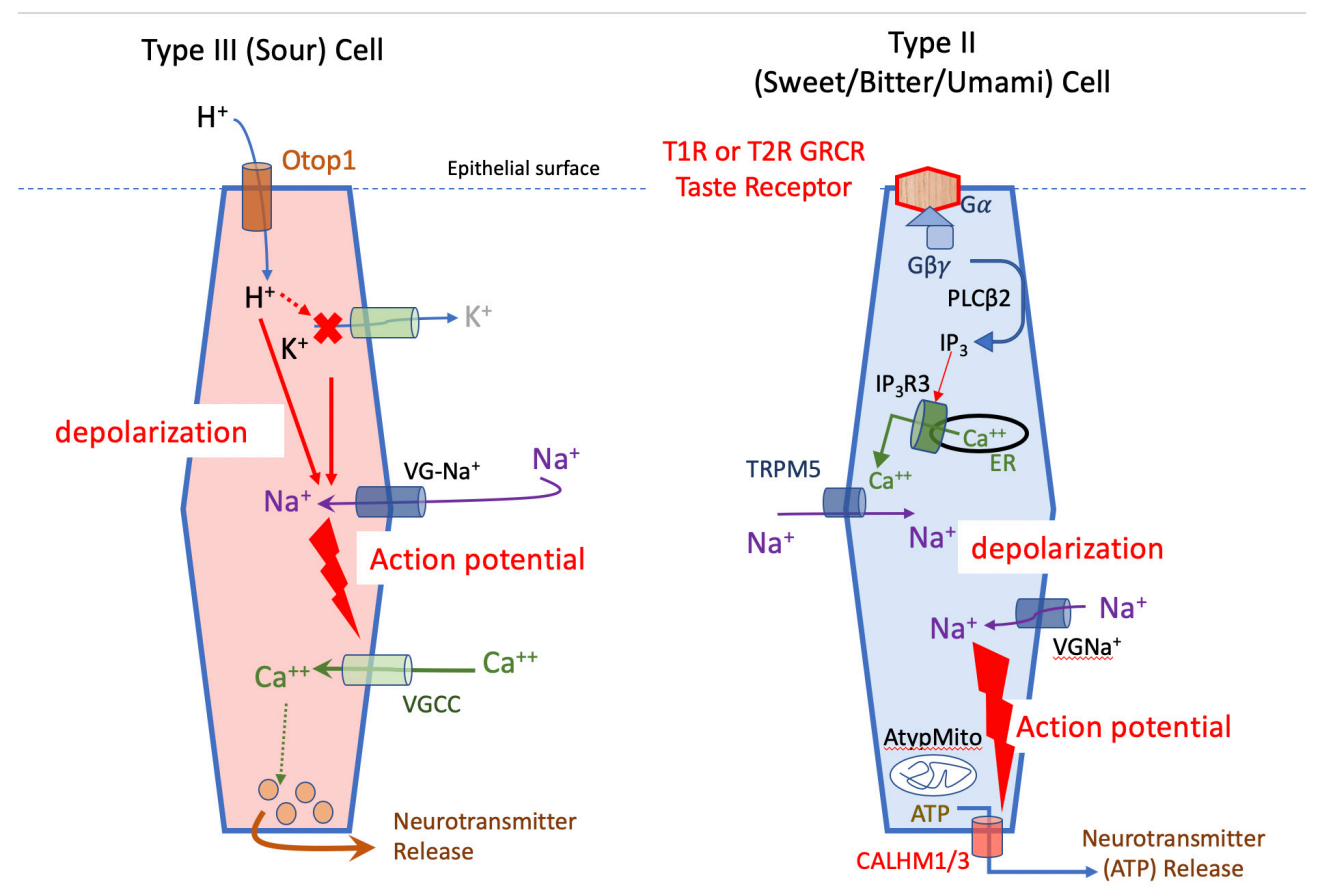
Taste transduction cascades. Transduction pathways for the two different types of taste-transducing cells: Type III for sour and Type II for sweet, bitter, or umami. The different responsiveness of Type II cells is dictated by the type of receptor each cell expresses, not by downstream members of the transduction cascade. AtypMito, atypical mitochondria; ER, endoplasmic reticulum; Gα, alpha subunit of G protein; Gβγ, beta-gamma subunits of G protein; IP _3_, inositol trisphosphate; IP _3_R3, inositol trisphosphate receptor isoform 3; PLCβ2, phospholipase C isoform β2; TRPM5, transient receptor potential cation channel subfamily M member 5; VGCC, voltage-gated calcium channel; VG-Na ^+^, voltage-gated sodium channel.

Although both Type II and Type III cells require action potentials for transmitter release, the mechanisms of release for these two cell types are quite different ( Figure 2). Type III cells use a conventional synapse involving voltage-gated Ca ^++^ channels and SNARE mechanisms to effect release of synaptic vesicles ^24, 46, 47^. In contrast, Type II taste cells (transducing bitter, sweet, or umami) rely on action potentials to trigger the voltage-gated large-pore channel CALHM1 to release ATP ^48– 50^. The pore size of this channel is sufficient to permit passage of ATP which serves as an obligatory transmitter in this system ^41, 51, 52^. The biophysical properties of CALHM1 as described in the seminal article on this channel ^48^ did not exactly match the properties of the release channel in taste buds. A more recent report ^49^ showed that the channel in taste buds consists of two subunits, CALHM1 and CALHM3, which together form a channel matching the properties of the Type II cell release channel.

Whereas the mechanism of release for ATP is well established for Type II cells, the source of ATP for transmission of sour information from Type III cells remains enigmatic. No one has yet demonstrated direct release of ATP from Type III cells, yet transmission of Type III cell taste qualities (for example, sour) is dependent on intact purinergic signaling to P2X receptors ^41^. Type III cells do not express CALHM1 ^48^ and so do not use CALHM1/3-mediated release. Another possible means of release of ATP is via synaptic vesicles, but Type III cells are reported to lack the vesicular ATP-transporter, VNUT ^53^, presumed to be necessary for loading of synaptic vesicles with ATP. So, what other possible sources exist for release of ATP? One suggestion is that Type III cells may trigger ATP release via interaction with other taste cell types. If so, this interaction does not require participation of Type II cells since mice lacking Type II cells (Skn1A-KO) ^54^ and CALHM1 KO ^48^ mice show nearly normal responses to sour. Hence, the transmission of sour information to taste nerves does not require the presence of Type II taste cells nor the function of CALHM1 to release ATP. Furthermore, a recent study showing high-resolution reconstructions of taste buds shows that Type III cells seldom directly contact Type II cells since processes from Type I cells intervene ^12^, suggesting that any interactions between Type II and Type III cells may be indirect.

## Tuning specificity of taste cells and nerve fibers

One of the classic discussions in taste research concerns the specificity of chemical tuning in the peripheral taste system, that is, whether individual taste cells or nerve fibers respond to single classes of taste stimuli or respond more broadly across several modalities. The former, known as “labeled line” coding, suggests that taste information (for example, for sweet taste) is transduced by a unique subset of taste cells and transmitted over dedicated nerve fibers that signal that particular quality (sweet) to the brain. Conversely, a broadly tuned peripheral system, utilizing “cross-fiber pattern” coding, relies on nerve fibers that are more broadly responsive but that respond maximally to particular qualities, necessitating comparison of activity across the fiber population in order for the brain to extract quality information. Receptor expression data strongly indicate that taste receptor cells largely express receptors for a single taste quality and this suggests that labeled line coding is plausible ^55^.

So, the question then is how specifically the taste cells activate particular nerve fibers. Two articles published in 2015 address this question but arrive at somewhat different conclusions, although detailed comparison of the data in the two studies shows similar results. In both works, the investigators used optical recording methods to assess activity in geniculate ganglion cells that innervate taste buds in fungiform papillae on the front of the tongue. Barretto *et al*. ^2^, using relatively low concentrations of taste stimuli, report that taste ganglion cells largely respond to a single stimulus class, although many multiply responsive cells are evident in the data. Conversely, Wu *et al*. ^3^ found that ganglion cell specificity is dominant only for low concentrations of tastants. As concentrations increase, more and more ganglion cells are recruited so that the majority of cells will respond to more than one class of tastant at moderate - high stimulus levels. This finding tends to support a cross-fiber model of decoding afferent input, particularly at higher stimulus concentrations. It should be noted that loss of specificity at high stimulus levels is similar to encoding in other sensory modalities; for example, a dim blue light of 505 nm will selectively activate the blue-sensitive, short-wavelength cones in the retina. But when the same wavelength of light is more intense, it will also activate the green, medium-wavelength cones and ultimately the red, long-wavelength cones. So absolute color cannot be determined by reading the output of only one class of cones but rather requires comparisons of activity levels across the different types of cones. Similarly, taste quality identification may require comparison of activity levels across incoming lines of information which preferentially, but not absolutely, encode particular qualities. In other words, while the afferent input is “tuned” as would be necessary for a labeled line system, absolute quality information can be extracted only by comparing the pattern and level of activity across fiber classes.

Another requirement for labeled line coding is connectional specificity between taste cells and nerve fibers; that is, sweet receptor-expressing taste cells should connect to nerve fibers receiving synaptic input only from other sweet receptor cells and so forth. Such specificity has been suggested recently for both Type III cells and Type II cells. Taste nerve fibers highly expressing the serotonin receptor 5-HT _3_ show preferential connectivity with Type III taste cells (sour) that accumulate and release serotonin ^9^. Argument for Type II cell connectional specification is based on transcriptome profiling of geniculate ganglion cells. Like dorsal root ganglion cells ^56^, geniculate ganglion cells fall into distinct molecular classes ^4^. Those ganglion cells that innervate taste buds express Phox2b, whereas those providing somatosensory innervation to the ear do not ^57^, so this transcription factor can be used to identify ganglion cells that innervate taste buds. Lee *et al*. ^10^ showed that connectional specificity for Type II cells is maintained by molecular encoding of receptor cell and nerve fiber identities by SEMA family guidance molecules. Likewise, Zhang *et al*. ^8^ found that genetic deletion of particular gene products correlates with loss of behavioral response to particular tastants— *Cdh4* for umami, *Cdh13* for bitter, and *Egr2* for salty—suggesting that these factors may serve to identify particular classes of gustatory ganglion cells (see Table 1). Since these proteins are expressed widely in the CNS (including in taste-processing areas), it is unclear whether the reported behavioral changes are attributable to changes in ganglion cell functionality or changes higher in the neuraxis. Furthermore, a more recent meta-analysis of these and other transcriptome data on ganglion cell subclasses fails to support the segregation of geniculate ganglion cell subtypes according to expression of these cadherins ^11^. Whether this all equates to absolute functional specificity of the taste neurons remains open to question. Substantial evidence exists for the possibility of cell-to-cell communication in taste buds ^1, 58^, and side-band activity in the taste nerves might result from such interactions between taste cells rather than direct convergence of input onto single fibers.

Another interesting outcome of the molecular profiling of the geniculate ganglion neurons innervating taste buds is the identification of a population expressing *Piezo2*, a marker of touch-sensitive ganglion cells ^4^. Such touch sensitivity of a subpopulation of gustatory ganglion cells would explain the residual tactile responsiveness of the gustatory nerves following genetic deletion of P2X receptors necessary for transmission from taste cells to nerve fibers ^41^. Thus, the nerve fibers themselves may be touch-sensitive and require no activation from the taste buds for tactile activation.

## Is “fatty” a taste?

A classic question in the field of taste research is “How many primary tastes are there?” In the 20th century, the debate focused on whether umami (savory) was a primary taste distinct from the classic qualities of salty, sour, sweet, and bitter. The discovery of a distinct molecular receptor for glutamate, coupled with the identification of glutamate (umami)-specific fibers in gustatory nerves and behavioral experiments showing the discriminability of glutamate from other tastes, clinched the case that umami is indeed a distinct primary taste. In the last few decades, the question of whether the taste of fat is a primary taste quality has been debated ^59^. Potential fat receptors GPR120 and CD36 were identified molecularly in taste buds about a decade ago ^60, 61^. But the mere presence of a receptor does not necessarily equate to the existence of a separate coding channel as would be expected of a primary taste quality. This year, Ninomiya *et al*. ^62^ identified, for the first time, a population of nerve fibers (F-fibers) in the chorda tympani nerve that respond uniquely to a fatty acid, linoleic acid, lending further credence to the idea that fat is a unique taste quality. Complicating the situation is that some of the F-fibers also show responses to glutamate, and although mice can be trained to recognize the taste of linoleic acid, they confound it with glutamate (see also 63), suggesting that fatty taste sensations may be intermingled with those for umami. Thus, the question remains as to whether fatty acid taste is a distinct primary taste or more a modulator of other taste qualities.

## Remaining unanswered questions

- What mechanisms and cells underlie transduction of AI salt taste, and what cells are responsible for AS salt taste?
- What is the source of ATP required for transmission of information from Type III (sour) cells to the afferent nerves?
- How much intercellular communication occurs within taste buds, and how does this affect the nature of the signal transmitted to the nerve fibers?
- Is fatty a primary taste quality? And how many other unrecognized primary taste qualities may exist?
